# Phenotypic and Genotypic Characterization of *Staphylococcus aureus* Isolated from Patients with Chronic Furunculosis and Osteomyelitis from Northwestern Poland

**DOI:** 10.3390/pathogens14090923

**Published:** 2025-09-12

**Authors:** Aleksandra Wcisłek, Joanna Jursa-Kulesza, Helena Masiuk, Bartłomiej Grygorcewicz, Beata Hukowska-Szematowicz, Piotr Prowans, Paweł Ziętek, Danuta Kosik-Bogacka

**Affiliations:** 1 Independent Medical Microbiology Laboratory, Pomeranian Medical University in Szczecin, Powstanców Wielkopolskich 72, 70-204 Szczecin, Poland; aleksandra.wcislek@pum.edu.pl (A.W.); joanna.jursa.kulesza@pum.edu.pl (J.J.-K.); helena.masiuk@pum.edu.pl (H.M.); 2 Department of Genomics and Forensic Genetics, Pomeranian Medical University in Szczecin, Powstanców Wielkopolskich 72, 70-204 Szczecin, Poland; 3 Institute of Biology, Laboratory of Infectious Biology, Molecular Biology and Immunology, University of Szczecin, Wąska 13, 71-412 Szczecin, Poland; beata.hukowska-szematowicz@usz.edu.pl; 4 Molecular Biology and Biotechnology Center, University of Szczecin, Wąska 13, 71-412 Szczecin, Poland; 5 Department of Plastic, Endocrine and General Surgery, Pomeranian Medical University in Szczecin, 1 Unii Lubelskiej Street, 71-252 Szczecin, Poland; piotr.prowans@pum.edu.pl; 6 Autonomous Knee Surgery Unit, Pomeranian Medical University in Szczecin, 1 Unii Lubelskiej Street, 71-252 Szczecin, Poland; pawel.zietek@pum.edu.pl; 7 Department of Biology, Parasitology and Pharmaceutical Botany, Pomeranian Medical University in Szczecin, Powstanców Wielkopolskich 72, 70-204 Szczecin, Poland

**Keywords:** *Staphylococcus aureus* osteomyelitis, *S. aureus* furunculosis, virulence factors, chronic infections, MRSA, MSSA, biofilm formation

## Abstract

*Staphylococcus aureus*, a Gram-positive coccus commonly found in the environment, is indeed a common cause of both superficial and deep infections. The aim of the study was to determine the virulence factors of *S. aureus* characteristic of chronic infections, including chronic furunculosis and chronic osteomyelitis. Phenotypic characteristics of the bacteria (ability to produce hemolysis, clumping factor, and coagulase; antibiotic susceptibility) and genotypic characteristics (presence of genes responsible for the production of enzymes and toxins; ability to form biofilm) were examined. The real-time PCR method was used to determine the presence of virulence genes. Biofilm production was confirmed using the crystal violet method. Antibiotic and chemotherapeutic susceptibility tests were performed using the disk diffusion method. In 90% of cases, *S. aureus* strains possessed the following virulence genes: clfA, clfB, spa, cna, eap, hlgA, hlgB, hlg, hld, bap, bbp, ebpS, fib, fnbA, fnbB, and pvl. A total of 82% of *S. aureus* strains showed susceptibility to methicillin (MSSA), whereas 12% of strains were susceptible to methicillin and simultaneously resistant to macrolides, lincosamides, and streptogramin B, including 10.5% with inducible resistance and 1.5% with constitutive resistance (MSSA/MLSB). In addition, 1.5% were methicillin-resistant *S. aureus* (MRSA) and susceptible to the remaining antimicrobial agents. The predominance of MSSA in the etiology of chronic furunculosis and chronic osteomyelitis was observed. It has been demonstrated that MSSA possesses a similar set of virulence genes to MRSA and that MSSA is responsible for most cases of chronic osteomyelitis and furunculosis. The findings indicate that *S. aureus* possesses numerous virulence factors that play a key role in the processes of adhesion to and proliferation within host cells.

## 1. Introduction

*Staphylococcus aureus* is a Gram-positive, facultative anaerobic bacterium [1], which colonizes the prenares of approximately 20–30% of healthy individuals and up to 40–50% of healthcare personnel, who can act as reservoirs and vectors for transmission in clinical settings [2]. This opportunistic pathogen can cause multiple human infections, such as impetigo, folliculitis, furuncles, carbuncles and cellulitis, toxin-mediated diseases, such as food poisoning, scalded skin syndrome, and toxic shock syndrome, and systemic and life-threatening conditions for endocarditis, pulmonary infections (e.g., necrotizing pnuemonia), deep-seated abscesses, meningitis, urinary tract, and bloodstream infections [3].

The mortality rate of *S. aureus* bloodstream infections reaches 30% [4] and most commonly is associated with the emergence of methicillin-resistant *S. aureus* (MRSA) strains, representing a therapeutic challenge. MRSA is prevalent in several hospitals (healthcare-associated methicillin-resistant *S. aureus*, HA-MRSA), particularly in Europe, Asia, and the United States. A significant concern is the high level of resistance among strains isolated from hospital-acquired infections to antibiotics and chemotherapeutics, including tetracyclines, aminoglycosides, macrolides, and lincosamides. In addition, these strains frequently exhibit resistance to fluoroquinolones, chloramphenicol, trimethoprim/sulfamethoxazole, fusidic acid, and rifampicin [5]. The World Health Organization and the Centers for Disease Control and Prevention continuously monitor the increasing resistance of *S. aureus* to antibiotics and chemotherapeutics [6,7]. Community-associated MRSA (CA-MRSA) strains are generally characterized by higher virulence but lower antimicrobial resistance and often infect individuals without significant predisposing risk factors for infection [8].

Methicillin-susceptible *S. aureus* (MSSA) is an important etiological agent of dermatitis and soft-tissue inflammation, including chronic furunculosis and chronic osteomyelitis. MSSA strains frequently exhibit higher virulence compared to MRSA, as the acquisition and maintenance of antibiotic resistance determinants in MRSA impose a metabolic fitness cost that may attenuate its pathogenic potential. Nevertheless, MSSA demonstrates numerous additional traits that confer enhanced resilience against environmental stressors [5,9]. One such characteristic is the ability to produce biofilm [10]. By protecting bacterial cells, biofilm formation significantly reduces the effectiveness of antibiotics and/or chemotherapeutics. Treatment of chronic *S. aureus* infections is challenging, and the pathogenesis of these infections is still poorly understood. The problem of chronic infections concerns patients diagnosed with metabolic diseases, including diabetes, as well as patients after orthopedic procedures in which *S. aureus* infection has occurred [11]. In some patients, the lack of clinically significant impairments that could affect the chronic nature of the inflammatory process was found. In the available literature, there is a limited number of *S. aureus* infections differentiated in terms of their clinical course. Therefore, the aim of the study was to determine the virulence factors of *S. aureus* that contribute to chronic infections.

*Staphylococcus aureus* isolated from chronic furunculosis and chronic osteomyelitis were selected for analysis. Accordingly, phenotypic characteristics of the bacteria (ability to produce hemolysis, clumping factor, and coagulase; antibiotic susceptibility) and genotypic characteristics (presence of genes responsible for the production of enzymes and toxins; ability to form biofilm) were examined. The analysis included genes encoding surface proteins (microbial surface components recognizing adhesive matrix molecules, MSCRAMM). These proteins are involved in adhesion to host tissues, weakening of the immune response, and promotion of biofilm formation. This group includes staphylococcal protein A (SpA), collagen-binding protein (Cna), fibronectin-binding proteins A (fnbA) and B (fnbB), and extracellular adherence protein (eap) [12,13]. Clumping factor A (ClfA) is the major staphylococcal fibrinogen (Fg)-binding protein that binds to fibrinogen in plasma, facilitating bacterial aggregation and immune evasion [14]. Other MSCRAMM components include elastin-binding protein (ebpS), fibrinogen-binding protein (Efb), and bone sialoprotein-binding protein (bbp). These structures can bind to molecules such as collagen (primarily via Cna), fibronectin (via FnbAB), and fibrinogen (via ClfAB and Fib), thereby enabling immune evasion and subsequent infection development [15]. Expression of the genes encoding these proteins is particularly elevated during the early stages of biofilm formation. A surface protein involved in biofilm production by *S. aureus* on all surfaces is biofilm-associated protein (Bap). In addition, genes responsible for toxin production were also included in the analysis. *S. aureus* produces and secretes multiple toxins with diverse roles in pathogenesis, particularly in immune evasion and modulation of the immune response [16]. These include four hemolysins, α (hlgA), β (hlgB), γ (hlg), and δ (hld), as well as Panton–Valentine leukocidin (PVL) [17,18].

## 2. Materials and Methods

### 2.1. Bacterial Isolates

In the present study, *S. aureus* strains (*n* = 67) from the collection of the Independent Medical Microbiology Laboratory of the Pomeranian Medical University in Szczecin (PUM) were analyzed. All strains were isolated from samples collected from boils and from bone with surrounding tissue excised during surgery in patients with chronic furunculosis (*n* = 43) and chronic osteomyelitis (*n* = 24) from northwestern Poland, respectively. The study was conducted in accordance with the Declaration of Helsinki and was approved by the Ethics Committee of Pomeranian Medical University (protocol code KB-0012/112/17, date 2 October 2017) for studies involving humans. The study was conducted between 2009 and 2020.

Analyzed strains were stored at −80 °C in tryptic soy broth (Becton Dickinson, Franklin Lakes, NJ, USA) supplemented with anhydrous glycerol (Chempur, Piekary Śląskie, Poland) at a volume ratio of 9:1. Before testing, the strains were subcultured onto Columbia agar enriched with 5% sheep blood (BioMérieux, Craponne, France) and incubated for 24 h at 37 °C. A three-phase streaking pattern was applied to ensure regular metabolic activity. Control *S. aureus* strains used in the experiments included ATCC 29213 and ATCC 6538 from the American Type Culture Collection (ATCC) [19,20].

### 2.2. Identification of Microorganisms

All strains grown on Columbia agar with 5% sheep blood exhibited beta-hemolysis. Species determination was conducted using biochemical tests (e.g., the Vitek 2 Compact System) (BioMérieux, Craponne, France) and basic preliminary microbiological diagnostic assays (e.g., agglutination tests and detection of the clumping factor, CF). The test for bound coagulase (clumping factor, CF) was performed using an agglutination test (Staphytect Plus, Oxoid, Basingstoke, UK) during the routine microbiological diagnostics.

### 2.3. Isolation of Chromosomal DNA

All strains were cultured onto Brain Heart Infusion (BHI) agar medium (BTL, Warsaw, Poland) and incubated at 37 °C for 18 h. DNA extraction was performed using the Genomic Mini AX Bacteria + SPIN kit (A&A Biotechnology, Gdańsk, Poland) according to the manufacturer’s instructions. Approximately 50–100 ng of genomic DNA per reaction was used.

Bacterial colonies from BHI agar were suspended in 1 mL of sterile water in an Eppendorf tube and vortexed vigorously for 30 s using a Vortex Lab Dancer (Binovo, Legnica, Poland). The tubes were then centrifuged at 13,000 rpm for one minute in an Eppendorf MiniSpin Plus centrifuge (Eppendorf SE, Hamburg, Germany). After centrifugation, the supernatant was discarded, and the sediment was resuspended in PBS buffer. Subsequently, 10 µL of lysozyme and 5 µL of mutanolysin were added to the suspension, which was incubated for 20 min at 50 °C in an Eppendorf Thermomixer 5436 (Eppendorf SE, Hamburg, Germany).

Following this, 20 µL of proteinase K and 200 µL of L1.4 buffer were added and mixed, and the mixture was incubated for an additional 10 min at 50 °C with vigorous shaking. The tubes were then vortexed for 20 s and centrifuged at 12,000 rpm for 45 s. Next, the supernatant was applied to Mini AX Spin columns and centrifuged at 12,000 rpm for 45 s. The columns were then transferred to 2 mL collection tubes included in the kit and washed sequentially with 600 µL of W1 buffer, followed by centrifugation at 12,000 rpm for 45 s, and then with 500 µL of W2 buffer, followed by centrifugation under the same conditions. Finally, the columns were transferred to 2 mL Eppendorf tubes, and DNA was eluted by adding 140 µL of elution buffer and centrifuging at 13,000 rpm for 60 s. The elution buffer combined with the isolated bacterial DNA settled at the bottom of the tube after centrifugation. Total bacterial DNA was used for subsequent stages of the study.

### 2.4. Real-Time PCR

A quantitative polymerase chain reaction (real-time PCR) technique was used to identify virulence genes in the bacterial genome. The primers were designed and made by Sigma-Aldrich (Merck, Poznań, Poland) based on [21,22,23,24] (Table 1). The primers in lyophilized form were dissolved in the manufacturer’s specified volume of sterile water, obtaining an initial concentration of 100 µmol. The next step was to obtain a working concentration of 10 µmol by diluting them in a ratio of 1:10. In this study, the following primers were used.

A real-time PCR kit (RT-HS PCR Mix SYBR A^®^, A&A Biotechnology, Gdańsk, Poland) was used in the study. The kit consisted of Master Mix and sterile water. The composition of Master Mix is shown below as follows:

- *Taq* DNA polymerase: 0.1 U/µL;
- MgCl_2_: 4 mM;
- dNTPs: 0.5 mM;
- 2 x reaction buffer with SYBR Green.

DNA was placed in a 96-well plate (Bio-Rad, Hercules, CA, USA), and then primers, sterile water, and Master Mix were added. The reaction mixture consisted (per sample) of the following:

➢ DNA: 1 µL;
➢ Starter F: 2 µL;
➢ R starter: 2 µL;
➢ Sterile water: 2.5 µL;
➢ Master Mix: 7.5 µL.

The PCR was carried out using the CFX Connect™ Real-time System (BioRad, Hercules, CA, USA), with thermal cycles tailored for each gene, ranging from 35 to 39 cycles depending on the virulence gene being amplified. For the staphylococcal protein A (spa), extracellular adherence protein (eap), clumping factor A (clfa) and B (clfb), collagen binding protein (cna), α- (hlgA), β- (hlgB), γ- (hlg), and δ- (hld) hemolysin genes, it was 39 cycles; for the Panton–Valentine leukocidin (pvl) gene, it was 38 cycles; and for the bone sialoprotein (bbp), penicillin-binding proteins (ebps), fibrinogen-binding protein (fib), fibronectin-binding protein a (fnpba) and b (fnpbb), biofilm-associated protein (bap) genes, it was 35 cycles. Thermal profile conditions are given in Table 2. Prior to the experiments, a rigorous calibration of the real-time PCR assays was conducted to ensure accuracy, involving primer specificity checks through gradient PCR and efficiency evaluations, with efficiencies confirmed between 90% and 110%. Calibration curves were then constructed for each gene from serial dilutions of template DNA to enable precise quantification of gene expression, correlating PCR cycle thresholds (Ct) with initial DNA amounts. Gene positivity was accurately determined if Ct values fell within the calibrated range of 15 to 35 cycles and were accompanied by distinct, specific melting peaks, ensuring the detection of the correct PCR products without nonspecific amplifications. Positive results were further validated using both no-template controls and positive controls containing known DNA amounts, reinforcing the reliability of our findings.

### 2.5. Biofilm Formation Ability of S. aureus Strains

The test of biofilm-generating capacity was conducted according to the procedure described by Kwiatkowski et al. [25]. Each strain was tested three times. The use of crystal violet in this study was dictated by the ability of this dye to stain live and dead bacteria and extracellular matrix products, as confirmed by Oleksy-Wawrzyniak et al. [26]. Similar studies were presented using crystal violet by Kamimura et al. [27] and Yang et al. [28]. In the first step, bacteria were resuspended in Tryptoy Soy Broth, TSB (Becton Dickinson, Franklin Lakes, NJ, USA) and then incubated at 37 °C for 18 h. The multiplied bacteria were diluted 1:200, placed in a 96-well plate in a hothouse at 37 °C, and incubated for 24 h. After incubation, the wells were washed 3 times with sterile saline solution (0.45% NaCl, bioMerieux, Craponne, France), fixed with methanol (Sigma Aldrich, Saint Louis, MO, USA) by adding 200 µL of this alcohol to each well, and left for 20 min at room temperature. The methanol was poured off, and the plate was left to dry. A total of 200 µL of 0.2% (*w*/*v*) crystal violet solution (Chempur, Piekary Śląskie, Poland) was poured into each well and incubated for 20 min at room temperature. The crystal violet was washed thoroughly by rinsing the wells 5 times with sterile distilled water. After rinsing, 200 µL of an 8:2 (*v*/*v*) ethanol/acetone mixture (P.P.H Stanlab, Gliwice, Poland; Chemland, Stargard, Poland) was poured in, and absorbance was read using an Envision 2104 Multi Reader (Perkin Elmer, Shelton, CT, USA) at λ = 590 nm. The control was three wells with crystal violet without bacteria. The results from the three measurements were averaged, and the value from the control test was subtracted from the individual results.

### 2.6. Determination of S. aureus Sensitivity to Antibiotics

Antibiotic susceptibility of *S. aureus* was evaluated according to European Committee on Antimicrobial Susceptibility Testing (EUCAST) guidelines. A 24 h bacterial culture was suspended in saline solution (0.9% NaCl) to obtain a concentration of 0.5 MacFarland turbidity standard. Bacterial suspension was inoculated onto Mueller–Hinton medium (BioMerieux, Craponne, France), where antibiotic discs were applied. The discs contained cefoxitin (30 µg), erythromycin (15 µg), gentamicin (10 µg), clindamycin (2 µg), ciprofloxacin (5 µg), and cotrimoxazole 25 µg (trimethoprim and sulfamethoxazole: 23.75 µg and 1.25 µg, respectively). The antibiograms were incubated for 20 h at 37 °C, and then the zone of inhibition was measured.

### 2.7. Statistical Analysis of the Results

Statistical analysis of the results was carried out using R version 4.1.1. A chi-square test was used to determine the relationship between the occurrence of a particular resistance phenotype and the induction of a specific infection. The same test was used to analyze gene occurrence versus resistance phenotype. The Kruskal–Wallis test analyzed the relationship between phenotypic resistance and biofilm production.

## 3. Results

### 3.1. Phenotypic Assessments

**Figure 1:**
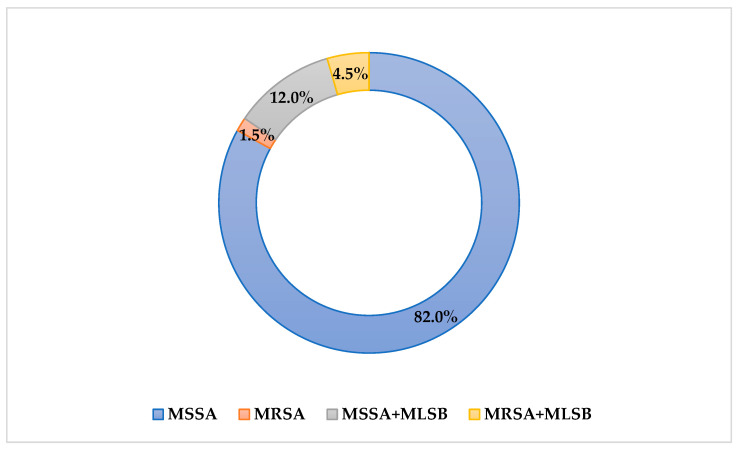
Percentage distribution of resistance phenotypes of the tested *S. aureus* strains.

Based on EUCAST guidelines, 82% of *S. aureus* strains tested showed methicillin susceptibility, while 12% of strains showed methicillin susceptibility with concomitant resistance to macrolides, lincosamides, and streptogramin B, including 10.5% with inducible resistance and 1.5% with constitutive resistance (MSSA/MLS_B_) (Figure 1). Among the strains tested, 1.5% were MRSA, and in 4.5% of MRSA, constitutive resistance to macrolides, lincosamides, and streptogramin B (MRSA/cMLS_B_) was simultaneously found. The lack of resistance to gentamicin, ciprofloxacin, and cotrimoxazole among all examined strains was also confirmed.

Our results showed that MSSA strains were responsible for more than 80% of bone infections and cases of furunculosis. Using a chi-square test, we found that MSSA strains were more likely to cause furunculosis (*p* = 0.0001) and osteomyelitis (*p* = 0.0001). This may suggest that the clinical picture of these infections is not directly influenced by the susceptibility or resistance of *S. aureus* strains but most likely linked with the presence of virulence genes that determine production of particular components exacerbating inflammation. In six isolates, the genetic profile revealed the presence of all investigated genes, with the exception of a single missing gene in each case. Among this group, four isolates originated from bone infections and two from furunculosis, of which two strains showed methicillin sensitivity and one strain showed methicillin sensitivity with iMLS_B_ resistance. Since only three *S. aureus* strains from patients with chronic furunculosis showed the absence of virulence genes, it can be concluded that the strains isolated from furunculosis are more virulent.

Five *S. aureus* strains isolated from patients with osteomyelitis showed the absence of virulence genes. In particular, one strain lacked three virulence genes, including the *bbp*, *fnpbA*, and *fib* genes encoding bone sialoprotein-binding protein, fibronectin-binding protein, and fibrinogen-binding protein, respectively. This indicates that, in individual cases, the induction of inflammation by *S. aureus* may be a much more complex process and not directly related to the presence or absence of virulence genes. Other parameters influence inflammation, including the patient’s age and comorbidities.

All tested *S. aureus* strains showed the ability to produce biofilm at different levels. The Kruskal–Wallis test found that MSSA produced more biofilm (*p* = 0.0183), even though MSSA strains are highly variable in biofilm formation. *S. aureus* strains that were observed to lack single genes showed little increase in biomass. Only the strain from a patient with chronic furunculosis showed a substantial biomass gain, at 0.27.

**Figure 2:**
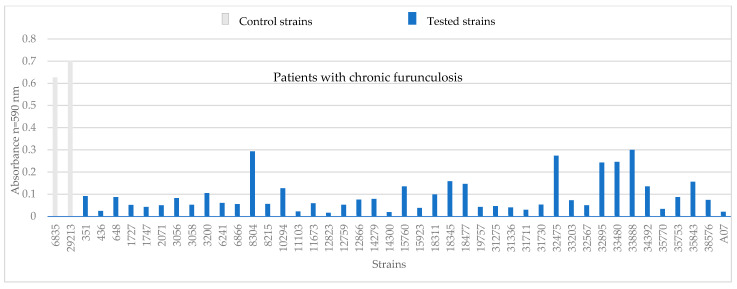
Absorbance value of *S. aureus* strains from patients with chronic furunculosis. Strains numbered 6538 and 29213 are control strains.

Tests conducted using crystal violet indicated that all tested *S. aureus* strains could produce biofilm. The absorbance value of the tested *S. aureus* strains ranged from 0.01 to 0.29. For *S. aureus* strains from patients with chronic furunculosis, a biomass increment ranging from 0.24 to 0.3 was observed. The highest biomass increment values were observed for five strains: 8304, 32475, 33203, 33480, and 33888 (Figure 2).

**Figure 3:**
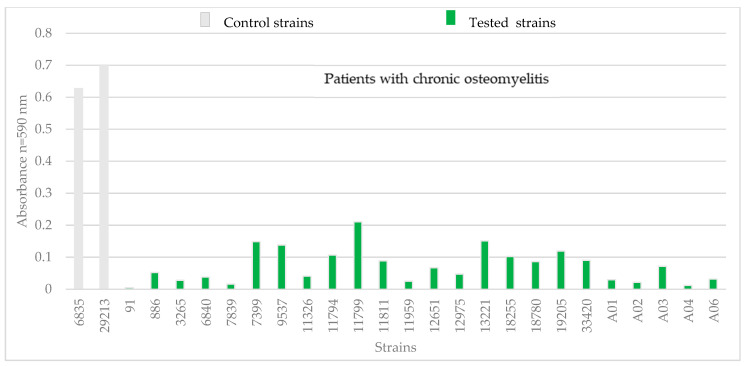
Absorbance value of *S. aureus* strains from patients with chronic osteomyelitis. Strains numbered 6538 and 29213 are control strains.

Among *S. aureus* strains isolated from patients with chronic osteomyelitis, four strains showed the greatest biomass increase: 11799, 7399, 13221, and 9537 (Figure 3). The biomass increment for these strains ranged from 0.14 to 0.21. The control *S. aureus* strains showed a higher biomass gain than the isolated *S. aureus* strains. The biomass gain of the two control strains was 0.6 and 0.7.

### 3.2. Genotypic Assessments

Real-time polymerase chain reaction studies showed that 59 strains possessed the following virulence genes: *clfa*, *clfb*, *spa*, *cna*, *eap*, *hlgA*, *hlgB*, *hlg*, *hld*, *bap*, *bbp*, *ebps*, *fib*, *fnpba*, *fnpbb*, and *pvl* (Table A1). Eight strains of *S. aureus* were found to be deficient in single genes, including five strains isolated from patients with chronic osteomyelitis and three strains isolated from patients with chronic furunculosis (Table 3). A chi-square analysis was conducted to investigate the potential relationship between various types of antibiotic resistance (MRSA, iMLSb, cMLSb) and the presence of specific virulence genes. The study included 16 virulence genes, namely, *clfa*, *clfb*, *spa*, *cna*, *eap*, *hlgA*, *hlgB*, *hlg*, *hld*, *bap*, *bbp*, *ebps*, *fib*, *fnpba*, *fnpbb*, and *pvl*. The analyses revealed no statistically significant associations between the resistances of MRSA, iMLSb, and cMLSb and the presence of most of the studied virulence genes, with *p*-values equal to 1 for each test.

These results suggest that the resistance and virulence mechanisms in *S. aureus* may operate independently, without direct links between these traits in the studied population. However, confirming these conclusions requires further research in diverse clinical and epidemiological settings to better understand the potential interactions between virulence genes and antibiotic resistance.

## 4. Discussion

In a study of *S. aureus* isolated from chronic osteomyelitis and furunculosis patients, over 80% of the strains were found to be sensitive to methicillin and other tested antibiotics. Approximately 10% of strains were methicillin-susceptible but exhibited resistance to macrolides, lincosamides, and streptogramin B, including 10.5% with inducible resistance and 1.5% with constitutive resistance. Additionally, 4.5% of strains were methicillin-resistant and showed constitutive resistance to macrolides, lincosamides, and streptogramin B, while 1.5% were methicillin-resistant without this combined resistance. All strains tested were susceptible to gentamicin, ciprofloxacin, and cotrimoxazole.

Studies on *S. aureus* isolated from pediatric patients with bacteremia and/or osteomyelitis identified approximately 77% of strains as methicillin-susceptible environmental isolates [29]. Gomes et al. [30] similarly found MSSA strains to be more prevalent than resistant strains across multiple inflammatory sites, including bone, and highlighted that MSSA infections can nonetheless pose a high risk for severe disease. An et al. [31] reported the isolation of both MRSA and MSSA strains (around 50% each) from bone infections in pediatric musculoskeletal cases, noting that hospitalization duration, outpatient treatment, and disease course were comparable regardless of methicillin resistance status. De la Calle et al. [32] observed that approximately 50% of pneumonia infections were caused by MSSA and 40% by MRSA, with mortality rates above 40% and 50%, respectively. In contrast, Klein et al. [33] found that hospitalization costs for MSSA and MRSA infections in the United States between 2010 and 2014 were similar, and in some cases, MSSA pneumonia treatment costs exceeded those for MRSA infections. Together, these findings from our study and the literature indicate that the infection risk and clinical burden of MSSA strains are comparable to, and in some cases exceed, those associated with MRSA.

Our findings underscore that MSSA strains, despite being methicillin-sensitive, can harbor virulence determinants comparable to MRSA, which is clinically relevant for infection management and antibiotic selection. Worth mentioning is the fact that MSSA can exhibit greater virulence than MRSA, a phenomenon attributed to the lower fitness costs associated with the absence of methicillin resistance. This allows MSSA strains to maintain more efficient growth, higher toxin production, and enhanced persistence in host tissues, which may contribute to the duration and severity of chronic infections [34,35].

Notably, the *cfiA* and *cfiB* genes, involved in iron acquisition, have been implicated in the persistence and chronicity of *S. aureus* infections. These genes facilitate bacterial survival in iron-limited environments, a common condition in chronic infections. Their presence in both MSSA and MRSA strains suggests that virulence potential, rather than methicillin resistance, may be a critical factor in the persistence of chronic infections [36].

The results presented by the ECDC [37] indicate a downward trend in the incidence of MRSA strains, decreasing from 19% in 2015 to 15.5% in 2019. In the present study, we demonstrated that chronic furunculosis and chronic osteomyelitis caused by MSSA occur more frequently than infections caused by resistant strains. Among the MSSA strains in our study, 12% exhibited constitutive and inducible resistance to macrolides, lincosamides, and streptogramin B (cMLSB and iMLSB phenotypes), while 4.5% of MRSA strains showed cMLSB resistance.

Saderi et al. [38] reported that *S. aureus* strains isolated from abscesses, wounds, and blood showed cMLSB phenotype in 92% of cases and an iMLSB phenotype in 6.3%. Pardo et al. [39] found that 22% of clinical isolates exhibited the cMLSB phenotype and 10% the iMLSB phenotype. In comparison, our study identified 10.5% of MSSA strains with the iMLSB phenotype and only 6% of both MRSA and MSSA strains with the cMLSB phenotype. These variations highlight that antibiotic resistance prevalence depends on the epidemiological context of specific regions and countries [40].

Therapeutic strategies should be based on antimicrobial susceptibility data provided by reference laboratories. In our study, the lack of resistance to cotrimoxazole, gentamicin, or ciprofloxacin was confirmed. Stein et al. [41] reported a treatment success rate using cotrimoxazole for bone infections caused by *S. aureus* in 66.7% of cases. Nguyen et al. [42] evaluated cotrimoxazole combined with rifampicin for osteomyelitis and infections related to orthopedic implants, finding a 78.6% efficacy compared to rifampicin with linezolid. All isolates examined in this study were susceptible to ciprofloxacin, though epidemiological data from the National Reference Center for Microbial Susceptibility (2019) indicate that approximately 15% of *S. aureus* strains resist fluoroquinolones. Due to the high percentage of resistant strains, quinolones should be considered as an alternative therapeutic option, used only after microbiological testing to ensure targeted therapy.

Boot et al. [43] explored the use of hyaluronic acid hydrogels loaded with gentamicin and vancomycin to treat infections associated with orthopedic implants, concluding that topical administration of gentamicin yielded the best therapeutic outcomes due to high local drug concentrations. Our study showed that *S. aureus* strains remain sensitive to gentamicin, suggesting promising therapeutic potential.

It has also been demonstrated that MSSA strains possess a similar repertoire of virulence genes as MRSA and are responsible for most cases of chronic osteomyelitis and furunculosis. Research indicates that *S. aureus* carries multiple virulence factors critical for adhesion and proliferation on host cells. Bacteria without antibiotic resistance demonstrate alternative strategies to withstand external environmental pressures [44]. One such mechanism is biofilm production, observed in both MSSA and MRSA strains, along with surface proteins that facilitate adhesion to host cells [45]. Biofilms protect bacterial cells, significantly impeding the effectiveness of antibiotics and chemotherapeutics.

In the present study, the analyzed *S. aureus* strains demonstrated a low capacity for biofilm production. Specifically, 62% of strains exhibited absorbance levels below 0.1, indicating limited biofilm formation. While this level is low, it likely provides sufficient protection against adverse environmental factors, including antibiotic exposure, as noted by Kavanagh et al. [44]. These authors emphasized the role of biofilms in chronic osteomyelitis, highlighting their contribution to the persistence and chronicity of inflammation. Bjarnsholt et al. [46] similarly noted that acute infections caused by planktonic bacteria are easier to treat than those involving biofilms. Biofilms are also implicated in other chronic conditions, such as chronic otitis and cystic fibrosis.

Akiyama et al. [47] isolated *S. aureus* from carbuncular lesions and cultured the bacteria on plasma- and fibrinogen-coated plates. Electron microscopy after 72 h revealed substantial fibrin deposition and increased bacterial proliferation as early as the fourth hour, underscoring the critical role of fibrinogen and fibrin in bacterial adhesion and biomass production. These findings suggest that *S. aureus* from furunculosis lesions is capable of biofilm formation.

Biofilm-associated bacteria can evade immune defenses by modulating host immune responses. Paharik and Horswill [48] demonstrated that macrophages near *S. aureus* biofilms show impaired phagocytic activity, with the *clfA* gene expression contributing to this inhibition. Moreover, *S. aureus* in biofilms can kill polymorphonuclear cells (PMNs), further evading phagocytosis and promoting chronic infection. Bhattacharya et. al. [49], corroborated by Paharik and Horswill [48], reported that high levels of Panton–Valentine leukocidin and hemolysins produced by *S. aureus* regulate NETosis, allowing bacteria to evade neutrophil attacks. Additionally, the production of polysaccharide intercellular adhesin (PIA) by *S. aureus* aids in avoiding neutrophil phagocytosis.

Recent insights into *S. aureus* biofilm formation show its crucial role in chronic infections and antibiotic resistance. Biofilms protect bacteria, hinder antibiotic penetration, and enhance tolerance by inducing metabolic dormancy and upregulating efflux pumps. Clinical isolates, especially MRSA, are increasingly resistant to vancomycin and daptomycin due to genetic mutations and biofilm-associated extracellular DNA. Small-colony variants (SCVs) in biofilms further complicate treatment by evading immune responses and antibiotics. Novel strategies like quorum-sensing inhibitors and biofilm-disrupting enzymes are being explored to tackle this resistance, highlighting the need for personalized therapies targeting both planktonic and biofilm states to reduce treatment failures [50,51].

Infections caused by biofilm-embedded *S. aureus* strains are challenging to treat, with therapies often failing in chronic cases even when strains demonstrate susceptibility to commonly used antimicrobial agents. Furthermore, bacteria within biofilms alter their prophylactic and metabolic profiles, resulting in resistance to antibiotics targeting the cell wall [48]. In this study, both MSSA and MRSA strains exhibited resistance to macrolides, lincosamides, and streptogramin B, supporting their capacity for biofilm-associated antibiotic resistance.

While significant efforts are being made to understand *S. aureus* virulence mechanisms and their roles in diseases such as osteomyelitis and furunculosis, further research is necessary to elucidate the inflammatory processes driven by these bacteria. The pathogenicity of *S. aureus* is closely related to the presence of various virulence genes [52]. In the presented study, all *S. aureus* strains isolated from patients with chronic furunculosis and chronic osteomyelitis were found to possess the following virulence genes: *clfa*, *clfb*, *spa*, *eap*, *hld*, *bap*, *ebps*, and *fnbb*. In contrast, 98.5% of the strains harbored the genes *hlgB*, *hlg*, *hlgA*, *pvl*, *cna*, and *fnbpa*, and 97% possessed *bbp* and *fib*. This study did not examine the allelic variant of bbp (sdrE), which may be present in their bbp-lacking strain. All *S. aureus* isolates from infected skin lesions of children with atopic dermatitis showed multiple virulence genes. The *hlgA*, *seu*, *fnbA*, *icaA*, and *sasG* genes were found in all isolates, *hlg* was found in 98.2% of isolates, and *cna* was found in 70.9% of isolates [53].

Li et al. [52] identified twelve virulence genes in *S. aureus* causing bloodstream infections, including *clfa* (100%) and *hlgA* (99%). It was found that *clfa* and *hlgA* were widely present and may play a pivotal role in the pathogenicity of *S. aureus*. This suggests that adhesion, proliferation, and subsequent development of inflammation by *S. aureus* strains are facilitated by the presence of multiple virulence factors.

In the present study, in all tested *S. aureus* strains, the spa gene encoding staphylococcal protein A (Spa) was present. This protein is crucial for typing *S. aureus* isolates. The spa gene encodes protein A, a critical virulence factor of *S. aureus*. Protein A binds to the Fc region of IgG antibodies, thereby disrupting opsonization and phagocytosis, which enables the bacteria to evade the host immune response. Additionally, protein A can interfere with immune signaling by interacting with tumor necrosis factor receptor 1 (TNFR1), modulating the inflammatory response. It also functions as an adhesin, facilitating bacterial attachment to host tissues. These combined actions contribute significantly to the pathogenicity of *S. aureus* and its ability to establish infections [54].

The significance of typing this gene using Based Upon Repeat Pattern (BURP) analysis in epidemiological studies was highlighted by Strommenger et al. [55]. In their study of *S. aureus* isolates from a reference center (*n* = 1459), they identified 221 different *spa* types among the strains studied. Many *S. aureus* strains have the ability to bind to fibronectin, as evidenced by the presence of the *fnbA* and *fnbB* genes. These genes have been found, among others, in *S. aureus* strains, causing infections associated with orthopedic implants. Giormezis et al. [56] demonstrated the presence of the *fnbA* gene in 88.9% of *S. aureus* strains isolated from various patients in three hospitals located in Greece.

The least understood gene is the *eap* gene, which encodes an extracellular adherence protein involved in binding the bacterial cell to the extracellular matrix of host tissues, making it crucial for colonization. In the present study, the *eap* gene was found in all tested *S. aureus* strains. Hussain et al. [57] examined 597 *S. aureus* isolates from humans and animals and found the *eap* gene in approximately 98% of strains.

Although the exact role of the extracellular adherence protein in the development of osteomyelitis and furunculosis is not fully understood, its ability to block monocytes and T cells indicates that this protein is not only involved in bacterial adhesion to host cells but also effectively inhibits the host immune response [58]. The collagen-binding protein, encoded by the *cna* gene, is directly linked to the development of osteomyelitis. This protein enables bacterial cells to bind to the host’s cartilage tissue and plays a key role in septic arthritis, endocarditis, and infections around orthopedic implants [59]. In the current study, one strain isolated from a patient with chronic osteomyelitis lacked the *cna* gene. Some previous studies have also reported the absence of this gene in strains still capable of causing infection and inflammation [59].

The biofilm-associated protein (Bap) is involved in biofilm production. In this study, all tested strains possessed the *bap* gene. Interestingly, this gene was present even in *S. aureus* strains that did not form strong biofilms. Most research on the *bap* gene has focused on *S. aureus* strains isolated from cattle, sheep, and goats. For example, Cucarella et al. [60] found the *bap* gene in only 5% of strains from cattle and did not detect it in human isolates (*n* = 75).

Cavalcante et al. [53] found that the bbp gene was significantly more present in *S. aureus* strains from infected skin lesions, with a *p*-value of 0.0021, suggesting a strong association between bbp and infection-related strains. Additionally, research by Bride et al. [61] indicated that the bbp gene was more prevalent in MSSA strains compared to methicillin-resistant strains, highlighting its potential role in the virulence of MSSA. These findings underscore the significance of the bbp gene in *S. aureus* pathogenicity, particularly in MSSA strains, and suggest that its presence may correlate with increased virulence.

In this study, three *S. aureus* strains from patients with chronic osteomyelitis lacked the *hlgA*, *hlgB*, and *hlg* genes, which encode alpha, beta, and gamma hemolysins, respectively. However, all tested strains possessed the *hld* gene. The gene most directly associated with furunculosis is *pvl*, which encodes Panton–Valentine leukocidin. Here, all but one strain carried this gene. Masiuk et al. [62] confirmed the role of Panton–Valentine leukocidin in skin and soft tissue infections, particularly furunculosis. The toxin’s ability to lyse leukocytes is a key factor in the inflammatory response.

PVL contributes to the chronicity of *S. aureus* infections by subtly undermining host defenses. By lysing neutrophils and releasing inflammatory mediators, PVL triggers tissue damage that creates niches for bacterial survival. This persistent inflammation, coupled with partial immune evasion, allows bacteria to persist and recur. Although PVL itself does not directly promote biofilms, the tissue damage it causes can facilitate their formation, further protecting bacteria from clearance and antibiotics. In essence, PVL drives a cycle of tissue injury and immune modulation that favors persistent and recurrent infections [63,64].

The *clfa* and *clfb* genes encode clumping factors A and B, which bind fibrinogen to host cells. In the present study, *clfa* and *clfb* were found in 99% and 100% of *S. aureus* strains, respectively. Similar findings were reported by Zmantar et al. [65], who detected *clfa* in 30% of strains isolated from patients with ear infections, along with several other virulence genes. In the study of Tristan et al. [66], the *clfa* gene was present in all *S. aureus* strains isolated from patients with endocarditis and in 95% of strains from patients with osteoarthritis. Similarly, the *clfb* gene was detected in all isolates from both patient groups.

Bone sialoprotein, encoded by the *bbp* gene, is related to fibrinogen α, a key component of bone and connective tissue. In the present study, 98.5% of *S. aureus* strains carried the *bbp* gene. The absence of *bbp* was observed in only one strain isolated from a patient with chronic furunculosis. In contrast, Nemati et al. [67] reported that *S. aureus* strains isolated from healthy and infected poultry lacked the *bbp* gene.

Tang et al. [26] used PCR to detect the *fib* gene in *S. aureus* strains from chickens, goats, and food poisoning samples, finding it present in over 90% of isolates. Similarly, Boden Wästfelt et al. [68] reported the *fib* gene in 97% of human *S. aureus* isolates and 100% of bovine isolates, using classical PCR methods. In our study, 97% of *S. aureus* strains were positive for the *fib* gene, consistent with previous findings. This suggests possible gene transmission among *S. aureus* strains, potentially increasing their virulence.

Additionally, the gene encoding elastin-binding protein (*ebps*) was detected in 100% of the tested *S. aureus* strains. Azmi et al. [42] found this gene in 84% of MRSA strains isolated from infected wounds, blood, urine, and nasal swabs using classical PCR. These findings underscore the crucial role of *ebps* in the *S. aureus* genome and its involvement in the inflammatory process. Overall, these findings highlight the complex interplay of antibiotic resistance, virulence factors, and biofilm formation in *S. aureus*, underscoring the critical need for targeted therapies guided by susceptibility testing to effectively manage chronic osteomyelitis and furunculosis.

Our study demonstrates that MSSA strains carry a diverse and abundant repertoire of virulence genes, highlighting their clinical significance in infection persistence and management. The significant biofilm production observed in MSSA, together with associations such as the *pvl* gene with furunculosis, underscores that virulence potential, rather than methicillin resistance alone, is a critical factor in chronic and acute *S. aureus* infections. These findings emphasize the need for careful monitoring of MSSA strains and consideration of virulence profiles in guiding therapeutic strategies.

This study is part of a larger project investigating the chronicity of *Staphylococcus aureus* infections, an area still underexplored. Our results show that MSSA strains carry a broad array of virulence genes, yet bacterial characteristics alone do not fully explain persistent infections. Chronicity appears closely linked to patient-specific factors, which vary widely and likely interact with bacterial traits. Future work will integrate patient-derived data and immune responses with strain characteristics to better understand the host–pathogen interactions driving long-lasting infections.

## Figures and Tables

**Table 1:** List of primers used in this study related to the experimental procedures.

Gene Name	Primer Sequence 5′-3′	Reference
*hlgA-F*	CTGATTACTATCCAAGAAATTCGATTG	
*hlgA-R*	CTTTCCAGCCTACTTTTTTATCAGT	[22]
*hlgB-F*	GTGCACTTACTGACAATAGTGC	
*hlgB-R*	GTTGATGAGTAGCTACCTTCAGT	[22]
*hld-F*	AAGAATTTTTATCTTAATTAAGGAAGGAGTG	
*hld-R*	TTAGTGAATTTGTTCACTGTGTCGA	[22]
*hlg-F*	GTCAYAGAGTCCATAATGCATTTAA	
*hlg-R*	CACCAAATGTATAGCCTAAAGTG	[22]
*spa-F*	CAGATAACAAATTAGCTGATAAAAACAT	
*spa-R*	CTAAGGCTAATGATAATCCACCAAATAC	[21]
*clfa-F*	ATTGGCGTGGCTTCAGTGCT	
*clfa-R*	CGTTTCTTCCGTAGTTGCATTTG	[21]
*clfb-F*	GCTGCAAAAATGCAAGATCA	
*clfb-R*	TTGCCGCCATAAATGTGTTA	[21]
*cna-F*	AAAGCGTTGCCTAGTGGAGA	
*cna-R*	AGTGCCTTCCCAAACCTTTT	[21]
*eap-F*	AGTCATTGATTACAACAA	
*eap-R*	CTTATTAAATGTTAAGCTTG	[21]
*bap-F* *bap-R*	GAGCCAAGACAAAGGTGAAG GTAGCCATAGCACGGAACAT	[24]
*fnba–F* *fnba–R*	TCCGCCGAACAACATACC TCAAGCACAAGGACCAAT	[24]
*fnbb–F* *fnbb–R*	TCTGCGTTATGAGGATTT ACAGTAGAGGAAAGTGG	[24]
*fib–F* *fib–R*	AGATGCGAGCGAAGGGTA TAAACGAAACTAAGTTGACTGC	[24]
*ebps–F* *ebps–R*	GGTGAACCTGAACCGTAG CTGGCAAGGCGAATAACT	[24]
*bbp–F* *bbp–R*	CTTAGCAGTTCAACAGGGTG TTGGCTTTATTGTGATGGTC	[24]
*pvl–F* *pvl-R*	AAATGCTGGACAAAACTTCTTGG TTTGCAGCGTTTTGTTTTCG	[23]

**Table 2:** Thermal profiles of the real-time PCR relations based on [21,22,23,24].

Stages	*spa, eap, clfb*	*cna, clfa*	*hlgA, hlgB, hld, hlg*	*Pvl*	*bbp, epbs, fib*	*fnbpa, fnbpb*	*Bap*
Initial denaturation	95 °C/ 5 min	95 °C/ 5 min	94 °C/ 7 min	94 °C/ 5 min	95 °C/ 5 min	95 °C/ 5 min	95 °C/ 5 min
Denaturation	95 °C/ 5 min	95 °C/ 5 min	94 °C/ 1 min	95 °C/ 15 s	95 °C/ 40 s	95 °C/ 40 s	95 °C/ 40 s
Annealing	50 °C/ 1 min	55 °C/ 1 min	58 °C/ 1 min	60 °C/ 30 s	56 °C/ 50 s	54 °C/ 50 s.	58 °C/ 50 s
Elongation	72 °C/ 1 min	72 °C/ 1 min	72 °C/ 1 min	60 °C/ 30 s	65 °C/ 5 s	65 °C/ 5 s.	65 °C/ 5 s
Final elongation	72 °C/ 10 min	72 °C/ 10 min	72 °C/ 7 min	72 °C/ 3 min	72 °C/ 50 s	72 °C/ 50 s.	72 °C/ 50 s
End of the process	72 °C/ 12 min	72 °C/ 12 min	72 °C/ 7 min	72 °C/ 10 min	72 °C/ 10 min	72 °C/ 10 min	72 °C/ 10 min

**Table 3:** Frequency of gene carriage among *Staphylococcus aureus* isolates from patients with chronic furunculosis and chronic osteomyelitis (*clfa*, clumping factor A; *clfb*, clumping factor B; *spa*, protein A; *cna*, collagen adhesin; *eap*, extracellular adherence protein; *hlgA*, alpha-hemolysin; *hlgB*, beta-hemolysin; *hlg*, gamma-hemolysin; *hld*, delta-hemolysin; *bap*, biofilm-associated protein; *bbp*, bone sialoprotein-binding protein; *ebpS*, elastin-binding protein; *Efb*, fibrinogen-binding protein; *fnbA*, fibronectin-binding protein A; *fnbB*, fibronectin-binding protein B; *pvl*, panton valentine leukocidin; +, indicates the presence of the gene in question; −, indicates an absence of the gene). The virulence genes in *S. aureus* isolates from patients with chronic furunculosis and chronic osteomyelitis. The table shows only those strains in which the presence of single genes was not observed (*cna*, collagen adhesin; *hlgA*, alpha-hemolysin; *hlgB*, beta-hemolysin; *hlg*, gamma-hemolysin; *hld*, delta-hemolysin; *bbp*, bone sialoprotein-binding protein; *fb*, fibrinogen-binding protein; *fnbB*, fibronectin-binding protein; *pvl*, panton valentine leucocidin).

	Gene Name	Osteomyelitis	Furunculosis
MSCRAMM	*Clfa*	100	100
	*Clfb*	100	100
	*spA*	100	100
	*Can*	95.8	100
	*Eap*	100	100
	*Bap*	100	100
	*Bbp*	100	97.7
	*ebpS*	100	100
	*fnbA*	100	100
	*fnbB*	100	97.7
	*Fib*	100	100
Cytotoxin	*hlgA*	95.8	100
	*HlgB*	95.8	100
	*Hlg*	95.8	100
	*Hld*	100	100
	*Pvl*	100	97.7

## Data Availability

The data presented in this study are available on request from the corresponding author.

## Appendix A

**Table A1:** The virulence genes in *Staphylococcus aureus* isolates from patients with chronic furunculosis and chronic osteomyelitis (*clfa*, clumping factor A; *clfb*, clumping factor B; *spa*, protein A; *cna*, collagen adhesin; *eap*, extracellular adherence protein; *hlgA*, alpha-hemolysin; *hlgB*, beta-hemolysin, *hlg*, gamma-hemolysin; *hld*, delta-hemolysin; *bap*; biofilm-associated protein; *bbp*, bone sialoprotein-binding protein; *ebpS*, elastin-binding protein; *Efb*, fibrinogen-binding protein; *fnbA*, fibronectin-binding protein A; *fnbB*, fibronectin-binding protein B; *pvl*, panton valentine leukocidin; +, indicates the presence of the gene in question; -, indicates an absence of the gene).

*Genes* Strain Number	*clfa*	*clfb*	*spa*	*cna*	*eap*	*hlgA*	*HlgB*	*hlg*	*Hld*	*bap*	*bbp*	*ebpS*	*Efb*	*fnbA*	*fnbB*	*Pvl*
control strains																
29213	+	+	+	+	+	+	+	+	+	+	+	+	+	+	+	+
6538	+	+	+	+	+	+	+	+	+	+	+	+	+	+	+	+
strains from patients with chronic osteomyelitis																
91	+	+	+	+	+	+	-	+	+	+	+	+	+	+	+	+
886	+	+	+	+	+	+	+	+	+	+	+	+	+	+	+	+
3265	+	+	+	+	+	+	+	+	+	+	+	+	+	+	+	+
6840	+	+	+	+	+	+	+	+	+	+	+	+	+	+	+	+
7839	+	+	+	+	+	+	+	+	+	+	+	+	+	+	+	+
7399	+	+	+	+	+	+	+	+	+	+	+	+	+	+	+	+
9537	+	+	+	+	+	+	+	+	+	+	+	+	+	+	+	+
11326	+	+	+	+	+	+	+	+	+	+	+	+	+	+	+	+
11794	+	+	+	+	+	+	+	+	+	+	+	+	+	+	+	+
11799	+	+	+	+	+	+	+	+	+	+	+	+	+	+	+	+
11811	+	+	+	+	+	+	+	+	+	+	+	+	+	+	+	+
11959	+	+	+	+	+	+	+	-	+	+	+	+	+	+	+	+
12651	+	+	+	+	+	+	+	+	+	+	+	+	+	+	+	+
12975	+	+	+	+	+	+	+	+	+	+	+	+	+	+	+	+
13221	+	+	+	+	+	+	+	+	+	+	+	+	+	+	+	+
18255	+	+	+	+	+	-	+	+	+	+	+	+	+	+	+	+
18780	+	+	+	+	+	+	+	+	+	+	+	+	+	+	+	+
19205	+	+	+	+	+	+	+	+	+	+	+	+	+	+	+	+
33420	+	+	+	+	+	+	+	+	+	+	+	+	+	+	+	+
40034	+	+	+	-	+	+	+	+	+	+	+	+	+	+	+	+
40675	+	+	+	+	+	+	+	+	+	+	+	+	+	+	+	+
41203	+	+	+	+	+	+	+	+	+	+	+	+	+	+	+	+
43846	+	+	+	+	+	+	+	+	+	+	+	+	+	+	+	+
43901	+	+	+	+	+	+	+	+	+	+	+	+	+	+	+	+
strains from patients with chronic furunculosis																
351	+	+	+	+	+	+	+	+	+	+	+	+	+	+	+	+
436	+	+	+	+	+	+	+	+	+	+	+	+	+	+	+	+
648	+	+	+	+	+	+	+	+	+	+	+	+	+	+	+	+
1727	+	+	+	+	+	+	+	+	+	+	+	+	+	+	+	+
1747	+	+	+	+	+	+	+	+	+	+	+	+	+	+	+	+
2071	+	+	+	+	+	+	+	+	+	+	+	+	+	+	+	+
3056	+	+	+	+	+	+	+	+	+	+	+	+	+	+	+	+
3058	+	+	+	+	+	+	+	+	+	+	+	+	+	+	+	+
3200	+	+	+	+	+	+	+	+	+	+	+	+	+	+	+	+
6241	+	+	+	+	+	+	+	+	+	+	+	+	+	+	+	+
6866	+	+	+	+	+	+	+	+	+	+	+	+	+	+	+	+
8304	+	+	+	+	+	+	+	+	+	+	+	+	+	+	+	+
8215	+	+	+	+	+	+	+	+	+	+	+	+	+	+	+	+
10294	+	+	+	+	+	+	+	+	+	+	+	+	+	+	+	+
11103	+	+	+	+	+	+	+	+	+	+	+	+	+	+	+	+
11673	+	+	+	+	+	+	+	+	+	+	+	+	+	+	+	+
12756	+	+	+	+	+	+	+	+	+	+	+	+	+	+	+	+
12823	+	+	+	+	+	+	+	+	+	+	+	+	+	+	+	+
12866	+	+	+	+	+	+	+	+	+	+	+	+	+	+	+	+
14279	+	+	+	+	+	+	+	+	+	+	+	+	+	+	+	-
14300	+	+	+	+	+	+	+	+	+	+	+	+	+	+	+	+
15760	+	+	+	+	+	+	+	+	+	+	+	+	+	+	+	+
15923	+	+	+	+	+	+	+	+	+	+	+	+	+	+	+	+
18311	+	+	+	+	+	+	+	+	+	+	+	+	+	+	+	+
18345	+	+	+	+	+	+	+	+	+	+	+	+	+	+	+	+
18477	+	+	+	+	+	+	+	+	+	+	+	+	+	+	+	+
19757	+	+	+	+	+	+	+	+	+	+	-	+	+	+	+	+
31136	+	+	+	+	+	+	+	+	+	+	+	+	+	+	+	+
31275	+	+	+	+	+	+	+	+	+	+	+	+	+	+	+	+
31711	+	+	+	+	+	+	+	+	+	+	+	+	+	+	+	+
31730	+	+	+	+	+	+	+	+	+	+	+	+	+	+	+	+
32475	+	+	+	+	+	+	+	+	+	+	+	+	+	+	-	+
32567	+	+	+	+	+	+	+	+	+	+	+	+	+	+	+	+
32895	+	+	+	+	+	+	+	+	+	+	+	+	+	+	+	+
33203	+	+	+	+	+	+	+	+	+	+	+	+	+	+	+	+
33480	+	+	+	+	+	+	+	+	+	+	+	+	+	+	+	+
33888	+	+	+	+	+	+	+	+	+	+	+	+	+	+	+	+
34392	+	+	+	+	+	+	+	+	+	+	+	+	+	+	+	+
35753	+	+	+	+	+	+	+	+	+	+	+	+	+	+	+	+
35770	+	+	+	+	+	+	+	+	+	+	+	+	+	+	+	+
35843	+	+	+	+	+	+	+	+	+	+	+	+	+	+	+	+
38576	+	+	+	+	+	+	+	+	+	+	+	+	+	+	+	+
45736	+	+	+	+	+	+	+	+	+	+	+	+	+	+	+	+
